# Linking African ancestral substructure to prostate cancer health disparities

**DOI:** 10.1038/s41598-023-47993-x

**Published:** 2023-11-27

**Authors:** Kazzem Gheybi, Naledi Mmekwa, Maphuti Tebogo Lebelo, Sean M. Patrick, Raymond Campbell, Mukudeni Nenzhelele, Pamela X. Y. Soh, Muvhulawa Obida, Massimo Loda, Joyce Shirindi, Eboneé N. Butler, Shingai B. A. Mutambirwa, M. S. Riana Bornman, Vanessa M. Hayes

**Affiliations:** 1 Ancestry and Health Genomics Laboratory, Charles Perkins Centre, School of Medical Sciences, Faculty of Medicine and Health, University of Sydney, Camperdown, NSW 2006 Australia; 2 School of Health Systems and Public Health, University of Pretoria, Pretoria, South Africa; 3 Department of Biochemistry, Genetics and Microbiology, University of Pretoria, Pretoria, South Africa; 4 Phulukisa Health Care, Pretoria, South Africa; 5 Tshilizini Hospital, Vhembe, Limpopo South Africa; 6 Department of Pathology and Laboratory Medicine, Weil Cornell Medicine, New York Presbyterian-Weill Cornell Campus, New York, NY USA; 7 Department of Epidemiology, University of North Carolina at Chapel Hill, Gillings School of Global Public Health, Chapel Hill, NC USA; 8 Department of Urology, Sefako Makgatho Health Science University, Dr George Mukhari Academic Hospital, Medunsa, South Africa; 9 Manchester Cancer Research Centre, University of Manchester, Manchester, M20 4GJ UK; 10 Faculty of Health Sciences, University of Limpopo, Turfloop Campus, Sovenga, Limpopo South Africa

**Keywords:** Risk factors, Urology

## Abstract

Prostate cancer (PCa) is a significant health burden in Sub-Saharan Africa, with mortality rates loosely linked to African ancestry. Yet studies aimed at identifying contributing risk factors are lacking within the continent and as such exclude for significant ancestral diversity. Here, we investigate a series of epidemiological demographic and lifestyle risk factors for 1387 men recruited as part of the multi-ethnic Southern African Prostate Cancer Study (SAPCS). We found poverty to be a decisive factor for disease grade and age at diagnosis, with other notably significant PCa associated risk factors including sexually transmitted diseases, erectile dysfunction, gynaecomastia, and vertex or complete pattern balding. Aligned with African American data, Black ethnicity showed significant risk for PCa diagnosis (OR = 1.44, 95% CI 1.05–2.00), and aggressive disease presentation (ISUP ≥ 4: OR = 2.25, 95% CI   1.49–3.40). New to this study, we demonstrate African ancestral population substructure associated PCa disparity, observing increased risk for advanced disease for the southern African Tsonga people (ISUP ≥ 4: OR = 3.43, 95% CI   1.62–7.27). Conversely, South African Coloured were less likely to be diagnosed with aggressive disease overall (ISUP ≥ 3: OR = 0.38, 95% 0.17–0.85). Understanding the basis for PCa health disparities calls for African inclusion, however, lack of available data has limited the power to begin discussions. Here, focusing on arguably the largest study of its kind for the African continent, we draw attention to the contribution of within African ancestral diversity as a contributing factor to PCa health disparities within the genetically diverse region of southern Africa.

## Introduction

Prostate cancer (PCa) has been shown to have a different course and essence in people of African ancestry. Not only is the transformation of the benign form to metastatic disease more precipitous, but tumor size is also reportedly larger, and African men present with more advanced disease^1,2^. PCa risk and disease course has also been associated with both rare and common African-specific inherited^3,4^, as well as cancer driver variants^5^. Additionally, several socioeconomic and environmental factors have been shown to contribute to higher PCa incidence and aggressiveness among African American^3^ and African continental populations^6^. While African American men are less likely to seek treatment for prostate-related disease, which has been contributed to a lack of insurance coverage, financial barriers and poor health-seeking attitudes^2,7^, dietary factors have also been considered^2^. It is therefore believed that the advanced form of the disease in African ancestral people stems from a combination of both genetic and non-genetic factors^8^.

Notably, PCa research pertaining to men of African ancestry has largely been driven out of the United States, with a notable lack of data from Sub-Saharan Africa. We have previously reported that Black men from South Africa are at 2.1-fold increased risk, after adjusting for age, to present with advanced PCa than African Americans from the National Cancer Institute (NCI) Surveillance, Epidemiology, and End Results (SEER) Program^9^. Within South Africa, this risk increased 1.6-fold for men living in subsistence farming over urban localities. Notably, the mortality rate of PCa in southern Africa is 2.7 times higher than the global estimates, with incidence rates on the increase due to improved screening and diagnosis^10^. However, factors mediating this pattern are still largely unknown, with a scarcity of studies on locally relevant lifestyle, demographic and environmental risk factors.

While it is well established that PCa risk and aggressive disease presentation is associated with African ancestry, what is unknown is if this contribution spans the spectrum of the genetically and culturally diverse African diaspora. The Southern African Prostate Cancer Study (SAPCS), recruiting within the borders of South Africa, provides a unique and currently unmet opportunity to address African-specific diversity, including four ancestrally (genetically and ethno-linguistically) distinct southern African populations, against non-African or ancestrally admixed South Africans. Interrogating a cohort of 1,387 southern African men diagnosed clinicopathologically either with or without PCa, we aimed to investigate the link between the rich southern African ancestral diversity and sociodemographic and environmental factors associated with PCa occurrence and advanced disease presentation.

## Materials and methods

### Study design

Initiated in 2008 in South Africa, the Southern African Prostate Cancer Study (SAPCS) aimed to identifying the genetic and non-genetic factors contributing to aggressive disease presentation across the region and associated ancestral disparity^6^. Irrespective of genetic ancestry or cultural heritage, study participants were recruited at one of multiple participating urology clinics located within the provinces of Limpopo including Polokwane Hospital or Tshilizini Hospital, and Gauteng including Steve Biko Hospital, Kalafong Hospital, Dr George Mukhari Academic Hospital between 2013 and 2019. The most common presenting complaints included urinary tract infections or erectile dysfunction. Patients with a recorded Gleason score (International Society of Urological Pathology (ISUP) group grading) or other records such as a biopsy indicating PCa, while radical prostatectomy and/or presence of metastasis were considered as cases and those with a definite diagnosis other than PCa or a negative biopsy were considered as controls. Patients whose PCa status could not be definitively determined were categorized as “unknown”.

### Study population

Excluding for non-South Africans, all men included in the study self-identified as representing one or more ethno-linguistic classifier. While data was collected for two generations, parental ethno-linguistic identifiers were used for participant classifications, with southern African identifiers including Ndebele, Pedi, Shangaan, Sotho (Northern), Swazi, Tswana, Tsonga, Venda, Xhosa, and Zulu (minus their ethnic or linguistic prefixes). Further classification using Guthrie classification of the Southern Bantu or zone S languages includes S20-Venda (Tshivenda speakers), S30-Sotho-Tswana (Sesotho, Sepedi, Setswana), S40-Nguni (isiNdebele, isiXhosa, isiZulu, siSwazi), and S50-Tsonga (Xitsonga, including in our study ethnically reported Shangaan). We have previously demonstrated that the Southern Bantu peoples have a distinct Bantu ancestral genetic heritage, which includes varied contributions of ancient KhoeSan contribution^11,12^. Non-Bantu speakers who self-identified as either Afrikaner, German, English South African, or White South African were classified in this study as European, while those who self-identified as Baster, Cape Coloured, Cape Malay or Indian were classified in our study as Admixed/Asian. We have previously published on the genetic ancestral substructure of the Baster of Namibia and the Cape Coloured of South Africa, which spanning over 12 generations (30 years a generation) has a historical link to European and Indian/south Asian ancestry through the Dutch East Indian settlement if the Cape, together with ancient KhoeSan and to a lesser degree Bantu genetic fractions^12^.

### Ancestral fractions

To further clarify the genetic ancestral fractions for the 780 Black South African participants, we performed ADMIXTURE v1.3.0 plot analysis^13^ using 33,410 single nucleotide polymorphisms (SNPs) pruned for linkage disequilibrium (LD), as previously described^14^ The ADMIXTURE analysis was conducted for K = 4, replicated in 3 of 10 runs, with a cross-validation error of 0.369. Ancestry proportions were plotted using the tool pong v1.5, with a greedy approach set to 0.95^15^.

### Sociodemographic data

Following the urology visit and evaluation, the sociodemographic data such as ethno-linguistic heritage were collected using a questionnaire. While patients were recruited within the Provinces of Limpopo and Gauteng, for each patient place of birth and previous (longer than 5 years) and current place of residence was recorded, observing a distribution of men having resided within all nine provinces of South Africa. Using the longest time of provincial residence, men were further classified according to poverty rate^16^ and included Eastern Cape and Limpopo, as high poverty rate; KwaZulu Natal, Mpumalanga and North West, as medium poverty rate; and Free State, Gauteng, Northern Cape and Western Cape, as low poverty rate. Provincial residence was also used to determine likely dependence on subsistence farming^17^, including Eastern Cape, Limpopo, North West and KwaZulu Natal. Occupations where lifting of no more than 10 pounds was required and involved sitting most of the times were listed as sedentary^18^. Jobs categorized as outdoor included driver, agriculture/gardening, defence, construction/mining. The type of balding, family history of PCa, history of diabetes and sexually transmitted disease (STD), aspirin use, gynaecomastia and erectile dysfunction were documented based on patient’s medical records and double-checked by the patient through trained SAPCS healthcare worker interviews. Traditional healers visit, having hairy chest and red meat consumptions were asked directly from the patients.

### Statistical analysis

Following defining the prevalence of the clinical and sociodemographic variables, we tested the association of each variable with PCa using logistic regression models and then adjusted for all the study variables (age, ethnicity, PCa family history, sexually transmitted disease history, red meat consumption, aspirin use, gynaecomastia, subsistence farming, poverty rate, traditional healers and occupation). This approach was taken to ensure that potential confounding factors were adequately addressed. To address the missing data on PCa status, the unknown group was included in the model once as cases and once as controls, allowing for a descriptive sensitivity analysis. This analysis explored how different assumptions regarding the unknown data could impact our findings.

In the analyses we had two categorizations for ethnicity. After incorporating all ethno-linguistic sub-categories, patients were then categorized as either Black South African or belonging to other groups, and the analysis was subsequently repeated. We further used logistic regression models to perform case only analysis for Gleason score and age at diagnosis. Age at diagnosis was also examined using an ordinal logistic regression. Lastly, we performed interaction term analysis with main effect between Black ethnicity and other variables to observe the differences of association with advanced PCa among Black South Africans and other ethnicities.

### Ethics statement

This study was approved by the research committee the University of Pretoria Faculty of Health Sciences Human Research Ethics Committee (HREC #43/2010, with US Federal wide assurance FWA00002567 and IRB00002235 IORG0001762) in South Africa, and as initially approved by the Department of Health and Social Development, Limpopo Provincial Government Ethics Committee (#001/2008) and University of Limpopo Medunsa Research and Ethics Committee (#MREC/H/28/2009). Further data interrogation performed under approval granted by the St. Vincent’s Sydney HREC (#SVH/15/227) in Australia. Informed consent was signed by all the individuals. Additional approval and review for the study was provided by the U.S. Army Medical Research and Materiel Command (USAMRDC), Office of Human and Animal Research Oversight (OHARO), Office of Human Research Oversight (OHRO), E02371. All experiments were performed in accordance with relevant guidelines and regulations.

## Results

**Figure 1:**
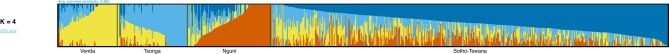
ADMIXTURE plot analysis for a representation of 780 Black South African men from the Southern African Prostate Cancer (SAPCS) for K = 4 (replicated in 3 of 10 runs, cross-validation error of 0.369), demonstrating a unique predominant genetic fraction distinguishing the African ancestral ethno-linguistic groups defined as Venda (yellow), Tsonga (light blue), Nguni (dark blue) and Sotho-Tswana (red).

Of the 1387 study participants visiting a contributing SAPCS urology clinic, 741 (53.4%) and 505 (36.4%) were identified as PCa cases and controls, respectively, while PCa status was undetermined for 141(10.2%) participants (Table 1). Among cases and controls 78.1% and 75.8% represented Black South African, 8.2% and 8.3% admixed/Asian and, 9.2% and 10.5% European ancestries, respectively, which is highly reflective of the population distributions across the country. Further determination of population substructure within the 780 SAPCS Black South Africans using ADMIXTURE analysis, concurring the existence of between ethno-linguistic genetic diversity (Fig. 1), which defines a predominant Venda (yellow), Tsonga (aqua), Nguni (red) and Sotho-Tswana (blue) genetic contributions and therefore within Southern Bantu population substructure. Furthermore, the age distribution across the cohort ranged from 40 to 107 (mean = 68.8). It was therefore not surprising that our study was biased towards pensioners, representing 74.0% and 70.7% of cases and controls, respectively. A total of 250 (33.7%) of the cases presented with a Gleason score of 8 or higher (ISUP ≥ 4).

**Table 1:** Sociodemographic and clinical data of the southern African study participants.

Variable			Control (%), n = 505	Case (%), n = 741	Unknown (%), n = 141
Age group		< 60	83 (16.4)	107 (14.4)	9 (6.4)
		60–67	163 (32.2)	204 (27.5)	49 (34.7)
		68–75	195 (38.5)	272 (36.7)	53 (37.6)
		75 +	62 (12.5)	156 (21.1)	30 (21.3)
		Missing	2 (0.4)	2 (0.3)	0
PSA		< 10	230 (45.5)	107 (14.4)	0
		10–20	198 (39.2)	118 (15.9)	0
		20–50	32 (6.3)	162 (21.9)	84 (59.6)
		50–100	5 (1.0)	103 (13.9)	23 (16.3)
		> 100	0	228 (30.8)	34 (24.1)
		Missing	40 (7.9)	23 (3.1)	0
Ethnicity	African/black	Nguni	59 (11.7)	124 (16.8)	26 (18.4)
		Sotho-Tswana	255 (50.6)	318 (42.8)	78 (55.3)
		Tsonga	30 (5.9)	62 (8.4)	12 (8.5)
		Venda	39 (7.7)	75 (10.1)	13 (9.2)
	Other/non-black	European	53 (10.5)	68 (9.2)	3 (2.1)
		Admixed	42 (8.3)	61 (8.2)	5 (3.6)
		Other	27 (5.3)	33 (4.5)	4 (2.8)
ISUP grade		1	–	216 (29.1)	–
		2	–	136 (18.4)	–
		3	–	114 (15.4)	–
		4	–	155 (20.9)	–
		5	–	95 (12.8)	–
		Missing	–	25 (3.4)	–
Poverty rate		Low	241 (47.7)	325 (43.9)	50 (35.4)
		Medium	59 (11.7)	107 (14.4)	19 (13.5)
		High	196 (38.8)	282 (38.1)	69 (48.9)
		Undefined	9 (1.8)	27 (3.6)	3 (2.1)
Subsistence farming		Yes	221 (43.8)	325 (43.9)	73 (51.8)
		No	275 (54.4)	389 (52.5)	65 (46.1)
		Undefined	9 (1.8)	27 (3.6)	3 (2.1)
Prostate cancer family history		Yes	48 (9.5)	81 (10.9)	13 (9.2)
		No	456 (90.3)	650 (87.7)	127 (90.1)
		Missing	1 (0.2)	10 (1.4)	1 (0.7)
Sexually transmitted diseases		Yes	159 (31.5)	247 (33.3)	59 (41.8)
		No	342 (67.7)	447 (60.3)	72 (51.1)
		Missing	4 (0.8)	47 (6.4)	10 (7.1)
Aspirin use		Yes	240 (47.5)	329 (44.4)	41 (29.1)
Having sedentary job		Yes	448 (88.7)	662 (89.3)	125 (88.7)
Having outdoor job		Yes	86 (17.0)	107 (14.4)	28 (19.7)
Gynaecomastia		Yes	68 (13.5)	130 (17.5)	27 (19.2)
Baldness pattern		Nil	173 (34.3)	207 (27.9)	53 (35.6)
		Frontal	96 (19.0)	117 (15.8)	24 (16.1)
		Vertex	25 (5.0)	55 (7.4)	9 (6.0)
		Complete	113 (22.4)	233 (31.4)	35 (23.5)
		Missing	98 (19.4)	129 (17.4)	20 (14.2)
Erectile dysfunction		Yes	304 (60.2)	460 (62.1)	92 (62.3)
		No	187 (37.0)	215 (29.0)	48 (34.0)
		Missing	14 (2.8)	66 (8.9)	1 (0.7)
Red meat consumption		Yes	313 (62.0)	470 (63.4)	98 (69.5)
		No	192 (38.0)	263 (35.5)	42 (29.8)
		Missing	0	8 (1.1)	1 (0.7)
Hairy chest		Yes	201 (40.4)	307 (41.4)	45 (30.2)
Diabetes		Yes	29 (5.7)	60 (8.1)	9 (6.4)
		No	333 (65.9)	487 (65.7)	89 (63.1)
		Missing	143 (28.3)	194 (26.2)	43 (30.5)
Traditional healers		Seek advice	131 (25.9)	202 (27.3)	35 (24.8)
		Don’t use	354 (70.1)	522 (70.5)	102 (72.3)
		Missing	20 (4.0)	17 (2.3)	4 (2.8)

### Case control and sensitivity analysis

**Figure 2:**
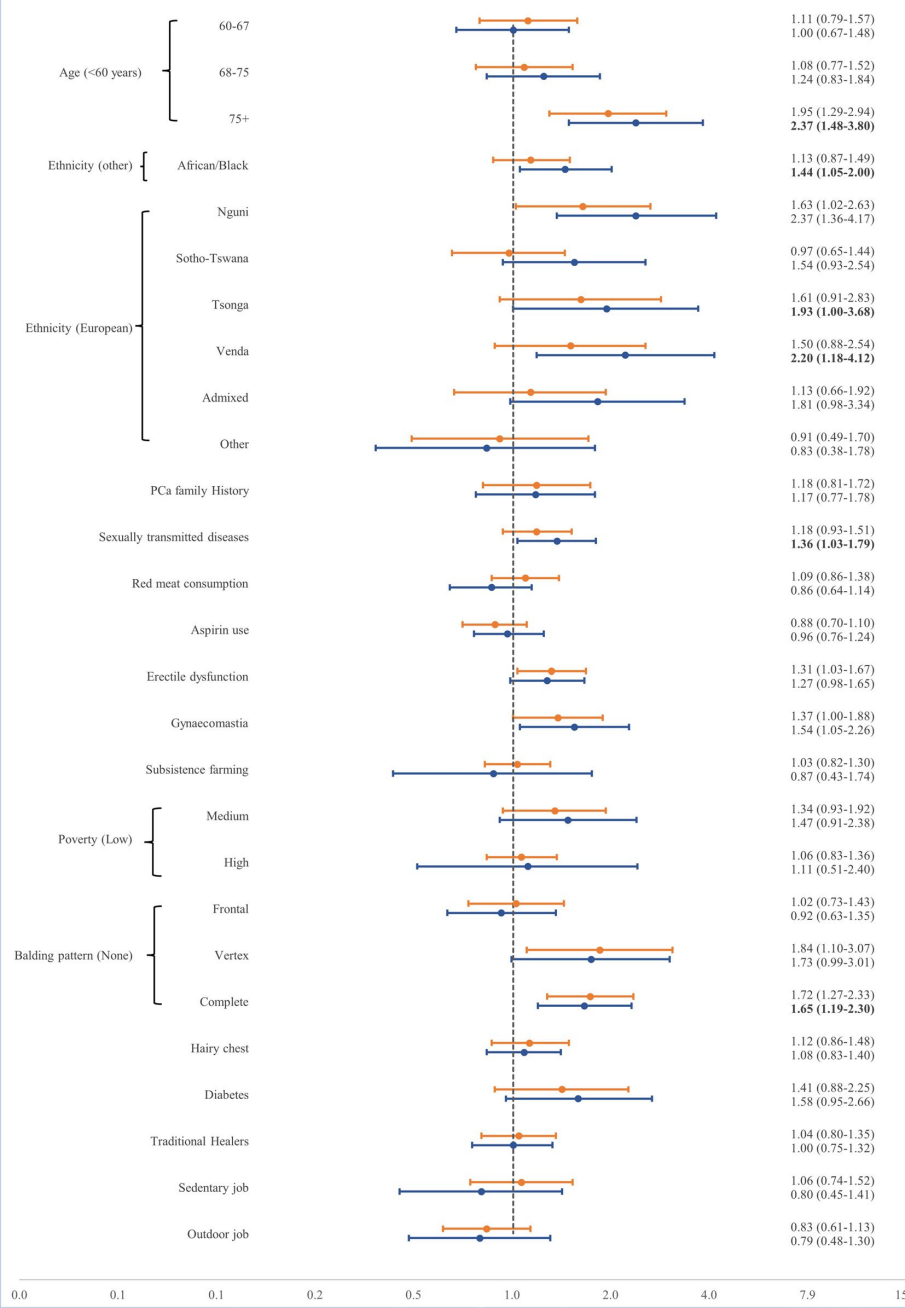
Crude (orange) and adjusted (blue) associations of study variables with the risk of prostate cancer using a logistic regression model for 741 southern African cases and 505 controls. Adjusted associations include age, ethnicity, PCa family history, sexually transmitted disease history, red meat consumption, aspirin use, gynaecomastia, subsistence farming, poverty rate, traditional healers and occupation. The X axis is based on odds ratios.

Given that we could not attain the PCa status of 141 participants, we have initially performed a complete case analysis (CCA). The CCA revealed that other than being a Nguni speaker (OR = 1.63, 95% CI 1.02–2.63), age of older than 75 years, erectile dysfunction, gynaecomastia, as well as vertex and complete balding pattern were associated with PCa (Fig. 2). The model was then adjusted for other variables, and it was shown that Black South Africans tend to be more likely than other ethnicities to be diagnosed with PCa (OR = 1.44, 95% CI 1.05–2.00). More specifically, Nguni, Tsonga, Venda and Admixed groups were more likely than those with a European ethnicity to be diagnosed with PCa. Other factors i.e. age of older than 75 years, history of STD, gynaecomastia and complete balding pattern were also associated with being diagnosed with PCa in this model. Performing a sensitivity analysis and assuming unknown individuals to be PCa cases didn’t change any significant results except for aspirin use (OR = 0.79, 95% CI 0.64–0.99). However, most of the variables including the ethno-linguistic variables lost the statistical significance assuming unknown individuals as controls (Table 1S).

### Grade analysis

**Figure 3:**
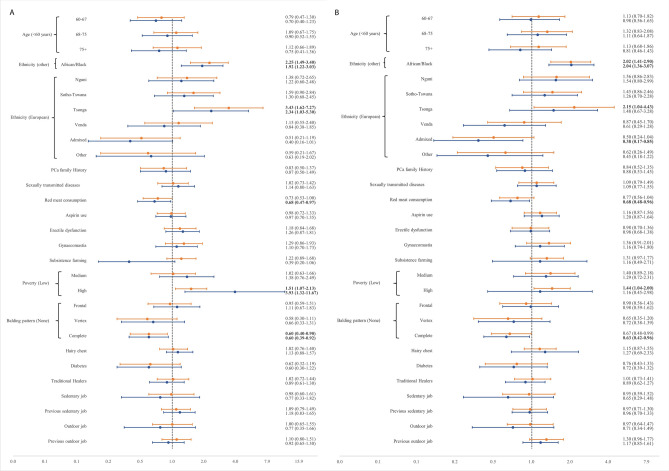
Association of study variables with ISUP ≥ 4 (**A**) and ISUP ≥ 3 (**B**) in unadjusted (orange) and adjusted (blue) logistic regression models for 716 southern African cases. Adjusted for age, ethnicity, PCa family history, sexually transmitted disease history, red meat consumption, aspirin use, gynaecomastia, subsistence farming, poverty rate, traditional healers and occupation. The X axis is based on odds ratios, *OR* odds ratio, *CI* confidence interval.

Black South Africans were more likely than all other groups to be diagnosed with an advanced disease (ISUP ≥ 4: OR = 2.25, 95% CI 1.49–3.40 and ISUP ≥ 3: OR = 2.02, 95% CI 1.41–2.90). In addition, even though all Black ethno-linguistic groups were more likely than Europeans to have advanced grade, this relation was significant only for the Tsonga people (ISUP ≥ 4: OR = 3.43, 95% CI 1.62–7.27). Residing in provinces with high poverty rates was also associated with advanced PCa grade presentation (ISUP ≥ 4: OR = 1.51, 95% CI 1.07–2.13). After adjusting with other variables, however still significant, the association of Black ethnicities and Tsonga people with ISUP ≥ 4 was moderated. Additionally, we found the Admixed/Asian less likely to be diagnosed with ISUP ≥ 3 compared with Europeans. People who had red meat consumption were also less likely to be diagnosed with advanced PCa (Fig. 3).

### Age at diagnosis

A logistic regression model showed that Black South African people were more likely than non-Africans to be diagnosed with PCa at an age greater than 59 years (OR = 1.64, 95% CI 1.04–2.60). In the ordinal logistic regression not only Black ethnicity, but all Black ethno-linguistic groups were associated with older age at diagnosis compared with Europeans. Those with a family history of PCa tended to be diagnosed with PCa at a younger age (Table 2S).

### Ethnicity

A positive family history of PCa was more likely to be found among non-Blacks than Black South Africans both in cases and the overall study population. Black people were more likely to live in provinces with subsistence farming or higher poverty rates, seek medical advice from traditional healers over first-call western practitioners, consume red meat, have gynaecomastia or a hairy chest, and report a previous outdoor job (Table 3S). Interaction term analysis showed that the pattern of association of age with advanced PCa (ISUP ≥ 4) is significantly different between Black South Africans and other ethnicities. Specifically, men older than 75 years of age were less likely to be diagnosed with advanced disease if they were of from Black South African group (OR = 0.21, 95% CI 0.04–0.98, Table 4S). The same pattern was observed in the association of red meat consumption (ISUP ≥ 4 and ISUP ≥ 3) and history of STD (ISUP ≥ 3) with advanced PCa. This shows that Black southern Africans had a significantly increased risk for the advanced disease compared to other ethnicities if they had a history of STD.

## Discussion

As no definite treatment exists for the metastatic PCa^9^, it is critical that PCa is detected early and pre-metastasis. As such, identifying risk factors for PCa and the advanced form of the disease in the region is an important step to decrease its burden on the health care system. The incidence of PCa in South Africa has tripled in the last 15 years, which has largely been attributed to improvements in diagnosis^19^. However, little is known about the tumor characteristics and the possible factors for this high incidence. Here we assessed for risk factors of PCa and its aggressiveness among the ancestries of southern African men.

Concurring with previous studies showing that Black South Africans are more likely to be diagnosed with PCa and with advanced disease^20^, including compared with African Americans^6^, we advocate for earlier age PSA screening (around 45 years) in the southern African setting, as suggested for African American men^21^. The association of the advanced disease with African over non-African ancestry remained statistically significant despite adjusting for known PCa risk variables, including age. Other than the genetic factors, an explanation for this association is that reportedly, only 9.9% of Black South Africans have private health insurance and are therefore reliant on often over-crowded and under-resourced public healthcare services; while 72.9% of Europeans, 52% of Indians and 17.1% of South African Coloured report having private health insurance^22^. Additionally, a recent study in South Africa of 341 PCa cases reported that only 76 (22.3%) had awareness of PCa before diagnosis, with less than 50% of cases seeking medical help after PCa diagnosis^23^, and as such we call for further programs focused on bringing education and awareness across the region.

However, identifying as a Black South African represents a rich ethno-linguistic and as such genetic and cultural diversity^24,25^, calling for caution in singularizing African ancestry within the region. This was highlighted in a 2017 study that showed the occurrence of malignancies to vary across different east African population identifiers^26^. Population classifiers aid in capturing the nuanced genetic variations and susceptibility to diseases, among different African subgroups. Using a smaller study population, we have previously shown a marginally increased PCa risk associated with the Venda Nation^9^. Here, through self-reported ethno-linguistic identification, we show the Nguni people to be statistically significantly more likely than Europeans to be diagnosed with PCa. Appreciating the limited number of cases, after adjusting the model for study variables we found Tsonga and Venda ethnicities were also associated with PCa. Furthermore, we found the Tsonga people were more likely to present with advanced form of the disease, after adjusting for age, providing for the first time within regional insights. Assuming genetic risk, we previously alluded to a differential ancestral Bantu fraction within the Tsonga versus Sotho-Tswana, while more closely reflecting the Venda peoples, while excluding for Nguni speakers in this initial analysis^6^. Through genetic population substructure analyses for the 780 Black South Africans self-identifying as Nguni, Sotho-Tswana, Tsonga or Venda speakers, we demonstrate unique population ancestral identifiers, with the Tsonga and Venda peoples representing more recent shared ancestral fractions. Besides shared genetics, the Tsonga in our study were largely recruited from the malaria endemic region of South Africa. Coinhabited by the Venda peoples, we have previously speculated on the potential impact of annual dichlorodiphenyltrichloroethane (DDT) spraying since the mid 1950s and associated with urogenital malformations in newborn Venda boys’ exposed in utero^27,28^ on PCa risk and aggressive disease^9^. Another possible explanation might be the frequent use of the medicinal plant “Xidomeja” (*J. Zeyheri*) by the Tsonga, which has been reported to contain diterpenoid used in synthetic vitamin E^29^. Notably, men using these supplements tend to be diagnosed with high-grade PCa^30,31^.

Besides African-specific ethno-linguistic identifiers, concurring with previous studies we associate gynaecomastia, erectile dysfunction and STDs with PCa risk^32,33^. While STDs were previously not associated with PCa risk in our smaller SAPCS study, gynaecomastia was associated with aggressive disease presentation, and erectile dysfunction associated with increased PCa risk and aggressive disease, including earlier onset of well differentiated tumours^9^. Aware that gynaecomastia and erectile dysfunction association can be due to increased patient age or PCa treatment^32^, notably, all SAPCS study participants were treatment naïve at time of recruitment, while the association of erectile dysfunction with PCa was no longer observed after adjusting for age. It is therefore highly likely that the older age of the cases is driving the positive correlation with erectile dysfunction. One must, however, caution that the controls in this study cannot be regarded as “healthy control” as most of them were elderly men with urological symptoms such as enlarged prostate or cystitis.

Additionally, we associate a high poverty rate with advanced disease. It is well-established that people with a lower income are less likely to use medical services^34^. Red meat consumption can be an indicative of better economic status and was inversely associated with the advanced disease. While in most African cultures red meat was consumed as part of ceremonial celebrations or coming together of families and communities, which contrasts with western cultures, specifically within South Africa where red meat is consumed daily^35^. Complete balding patterns was also inversely associated with advanced PCa, which although arguably converse to the inconsistent European-biased studies^36^, concurs with previous observations for Southern African men, including a decreased risk for advanced PCa further associated with younger age of balding^9^. While some testosterone inhibitors such as finasteride are used for curing baldness in men which also tend to lower PCa and its mortality rate^37^, this is unlikely to be commonly used within a less affluent study cohort. Age is usually the predictor of advanced-grade cancer where there is a national screening program for a specific age group^38,39^, hence associations between age and the advanced disease was not expected in this study.

Unlike many malignancies, PCa is usually a slow-progressing disease and as such it can be obscure for a long time before its diagnosis^40^. Black South Africans were diagnosed at an older age in this study. This again might reflect the poor health seeking attitude, reliance on traditional methods of health care, lack of screening and insurance coverage which causes a delay in PCa diagnosis in this population. However, this delay in diagnosis did not seem to be the cause of the advanced disease among Africans since the associations were still significant after adjusting for age. People with a positive family history of PCa being diagnosed at a younger age showed that there was probably a wariness in this population about PCa. In addition, PCa with a pathogenic genetic variant usually occurs at a younger age^41^. Some of the factors associated with an old-age diagnosis of PCa such as residing in subsistence farming areas are likely to reflect poor socioeconomic status. Since it is well-established that Black ethnicity is a risk factor for PCa^2^, the observations that Black South Africans are less likely to have a PCa family history, is suggestive that many Black South Africans remain undiagnosed.

The interaction term analysis was used to determine whether any of the study variables have different associations with advanced disease in Black southern Africans compared with non-African ethnicities. Red meat consumption was significantly a stronger predictor of advanced PCa in Black South Africans compared with others. This may stem from the fact that men of European ancestry are more likely to voluntarily choose vegetarianism, while red meat consumption better reflects the economic status for Black South Africans^42,43^. STDs also put Black men at greater risk for presenting with ISUP > 3 disease. In other words, a Black South African man is significantly more likely to be diagnosed with ISUP > 3 if he has an STD than a non-Black man with STD. This shows that in addition to screening for PCa, awareness should be raised among the Black community to preserve their sexual health. Age of older than 75 years was also a less decisive factor for being diagnosed with the advanced disease for African men, with ISUP group grades more akin to people younger than 60 years if they were Black South Africans.

The main strength of this study was the broad coverage of PCa cases over two provinces of South Africa and investigating some novel factors within arguably genetically one of the most diverse populations globally. Considering the generalizability of our findings, it’s important to note that our study sample, defined by specific inclusion criteria, may not perfectly mirror the broader population. We did exclude participants with unknown PCa status, but this group represented a small fraction of our sample. While our results remain robust and consistent, sensitivity analyses indicate that the associations are not solely dependent on our sample composition. As such, the inclusion of the sensitivity analysis for unknown cases provided some reassuring known PCa significances, for example Aspirin use reducing PCa risk^44^ and presence of STDs increasing risk^45^. While arguably under-powered compared to European-biased epidemiological studies, as the world recognises the importance for inclusivity and equity, specifically with regards to under-representation across the African diaspora, this study provides important insights as the largest regionally defined Sub-Saharan PCa study of its kind to date.

## Conclusion

In the era of personalized medicine, epidemiological factors often receive less attention than molecular factors and tumor characteristics. Here, we have shown that the Black South African ethno-linguistic identifier is associated with PCa and aggressiveness at diagnosis, regardless of other environmental factors, specifically and novel to this study highlighting increased risk for advanced disease in men from the Tsonga Nation. Additionally, we found poverty rate to be a decisive factor in being diagnosed with advanced PCa and delayed diagnosis. Our results confirms that men with an African ancestry should be encouraged to undergo earlier-aged PCa screening, with emphasis on preservation of sexual health and implementation of programs focused on PCa education and awareness. Complementary and parallel to genetic risk association studies focused on PCa health disparities, it is paramount that contributing non-genetic epidemiological risk factors are interrogated, especially as one considers the rich demographic, lifestyle and environmental diversity across the greater regions of Sub-Saharan Africa. The results of our investigation into risk factors within the African population provide a deeper understanding of the universality of certain risk factors associated with prostate cancer. This highlights the pressing need for greater inclusivity and equity in future prostate cancer studies.

### Supplementary Information

Supplementary Tables.

## Data Availability

The data underlying this article were obtained from the Southern African Prostate Cancer Study (SAPCS) and cannot be shared publicly due to the privacy of individuals who participated in the study. Researchers can apply for access to deidentified data through the SAPCS Data Access Committee management team V.M. Hayes, M.S.R. Bornman and/or S.B.A. Mutambirwa.
