# Supplementary material for: Linking African ancestral substructure to prostate cancer health disparities
Source: Sci Rep. 2023 Nov 27;13:20909. doi: 10.1038/s41598-023-47993-x (PMC10684577; doi:10.1038/s41598-023-47993-x)
Supplement: Supplementary file 1 — Supplementary Tables. [file 41598_2023_47993_MOESM1_ESM.pdf]

Linking African ancestral substructure to prostate cancer health disparities

Kazzem Gheybi, Naledi Mmekwa, Maphuti Tebogo Lebelo, Sean M. Patrick, Raymond Campbell, Mukudeni Nenzhelele, Joyce Shirindi, Pamela X.Y. Soh, Muvhulawa Obida, Massimo Loda, Eboneé N. Butler, Shingai B.A. Mutambirwa, M.S. Riana Bornman, and Vanessa M. Hayes

SUPPORTING DATA

Table 1S. Sensitivity analysis of the associations of study variables with the risk of prostate cancer in logistic regression models

**Variable (Base)**		**Sensitivity (case)** OR (95%CI)	**Sensitivity (control)** OR (95%CI)
**Age (<60 years)**	60-67	1.11 (0.79-1.57)	0.83 (0.58-1.16)
	68-75	1.19 (0.85-1.66)	0.94 (0.68-1.30)
	75+	**2.15 (1.43-3.21)**	1.46 (0.99-2.13)
**Ethnicity (other)**	African/Black	1.29 (0.99-1.68)	0.93 (0.72-1.21)
**Ethnicity (European)**	Nguni	**1.90 (1.19-3.02)**	1.20 (0.76-1.88)
	Sotho-Tswana	1.16 (0.78-1.71)	0.79 (0.53-1.15)
	Tsonga	**1.84 (1.05-3.20)**	1.21 (0.71-2.06)
	Venda	**1.68 (1.00-2.83)**	1.19 (0.72-1.96)
	Admixed	1.20 (0.70-2.04)	1.09 (0.65-1.83)
	Other	0.99 (0.54-1.81)	0.85 (0.46-1.55)
**PCa family History**		1.15 (0.80-1.65)	1.19 (0.83-1.69)
**Sexually transmitted diseases**		**1.26 (1.00-1.60)**	1.05 (0.84-1.31)
**Red meat consumption**		1.14 (0.91-1.43)	1.01 (0.81-1.27)
**Aspirin use**		**0.79 (0.64-0.99)**	1.03 (0.84-1.28)
**Erectile dysfunction**		**1.29 (1.02-1.63)**	**1.27 (1.01-1.59)**
**Gynaecomastia**		**1.42 (1.04-1.94)**	1.19 (0.89-1.60)
**Subsistence farming**		1.09 (0.87-1.36)	0.97 (0.77-1.20)
**Poverty (Low)**	Medium	1.37 (0.97-1.94)	1.23 (0.88-1.71)
	High	1.15 (0.91-1.46)	0.95 (0.76-1.20)
**Balding pattern (None)**	Frontal	0.98 (0.71-1.35)	1.06 (0.77-1.46)
	Vertex	**1.70 (1.03-2.81)**	**1.76 (1.10-2.82)**
	Complete	**1.57 (1.17-2.11)**	**1.72 (1.30-2.27)**
**Hairy chest**		1.15 (0.87-1.55)	0.98 (0.76-1.29)
**Diabetes**		1.37 (0.87-2.17)	1.37 (0.89-2.09)
**Traditional Healers**		1.02 (0.80-1.32)	1.06 (0.83-1.35)
**Sedentary job**		1.05 (0.74-1.49)	1.06 (0.76-1.49)
**Outdoor job**		0.89 (0.66-1.19)	0.79 (0.59-1.05)

Table 2S. Association of study variables with age at diagnosis in logistic and ordinal logistic regression models in prostate cancer cases.

Variable (Base)		Older than 59	Ordinal regression
**Ethnicity (other)**	African/Black	**1.64 (1.04-2.60)**	**2.19 (1.60-3.00)**
**Ethnicity (European)**	Nguni	1.55 (0.68-3.55)	**2.00 (1.19-3.38)**
	Sotho-Tswana	1.40 (0.69-2.82)	**2.20 (1.38-3.56)**
	Tsonga	1.68 (0.62-4.59)	**2.23 (1.20-4.13)**
	Venda	1.25 (0.51-3.04)	**3.06 (1.67-5.57)**
	Admixed	0.87 (0.36-2.12)	0.96 (0.52-1.75)
	Other	0.67 (0.24-1.84)	1.21 (0.58-2.55)
**PCa family History**		0.70 (0.38-1.28)	**0.64 (0.42-0.98)**
**Sexually transmitted diseases**		0.94 (0.60-1.48)	**0.71 (0.54-0.94)**
**Red meat consumption**		0.86 (0.55-1.33)	1.00 (0.76-1.32)
**Aspirin use**		1.12 (0.74-1.70)	0.98 (0.76-1.28)
**Erectile dysfunction**		1.15 (0.71-1.83)	1.03 (0.77-1.39)
**Gynaecomastia**		1.47 (0.81-2.68)	**1.76 (1.24-2.50)**
**Subsistence farming**		**1.71 (1.10-2.65)**	**2.30 (1.75-3.03)**
**Poverty (Low)**	Medium	1.39 (0.75-2.56)	1.19 (0.80-1.76)
	High	**2.12 (1.30-3.44)**	**2.91 (2.15-3.94)**
**Balding pattern (None)**	Frontal	1.20 (0.64-2.26)	0.93 (0.61-1.41)
	Vertex	1.84 (0.66-4.20)	1.26 (0.73-2.18)
	Complete	1.55 (0.90-2.67)	1.01 (0.72-1.42)
**Hairy chest**		0.91 (0.61-1.31)	0.83 (0.49-1.25)
**Diabetes**		1.51 (0.62-3.66)	0.87 (0.54-1.39)
**Traditional Healers**		1.14 (0.70-1.84)	1.30 (0.96-1.74)
**Sedentary job**		**3.26 (1.92-5.53)**	**2.28 (1.45-3.58)**
**Previous sedentary job**		0.96 (0.63-1.47)	**0.73 (0.56-0.96)**
**Outdoor job**		**0.24 (0.15-0.39)**	**0.27 (0.18-0.40)**
**Previous outdoor job**		1.48 (0.97-2.28)	**1.35 (1.03-1.77)**

Table 3S: Association of study variables with black South African ethnicity among all study participants and PCa cases

Variable (Base)		All individuals	Cases only
**PCa family History**		**0.51 (0.35-0.74)**	**0.49 (0.29-0.80)**
**Sexually transmitted diseases**		1.24 (0.92-1.66)	1.16 (0.76-1.77)
**Red meat consumption**		**1.90 (1.46-2.48)**	1.31 (0.91-1.88)
**Aspirin use**		0.81 (0.63-1.05)	0.90 (0.63-1.28)
**Erectile dysfunction**		1.05 (0.78-1.41)	0.93 (0.60-1.45)
**Gynaecomastia**		**3.90 (2.33-6.52)**	**2.90 (1.58-5.30)**
**Subsistence farming**		**3.04 (2.27-4.08)**	**2.07 (1.42-3.03)**
**Poverty (Low)**	Medium	1.44 (0.99-2.09)	1.58 (0.94-2.64)
	High	**6.21 (4.30-8.97)**	**3.62 (2.32-5.66)**
**Balding pattern (None)**	Frontal	**0.42 (0.28-0.63)**	**0.32 (0.18-0.60)**
	Vertex	0.69 (0.37-1.28)	0.66 (0.27-1.59)
	Complete	**0.66 (0.45-0.98)**	**0.44 (0.26-0.78)**
**Hairy chest**		**2.11 (1.47-3.05)**	**2.60 (1.46-4.65)**
**Diabetes**		**0.58 (0.36-0.93)**	0.66 (0.36-1.21)
**Traditional Healers**		**4.08 (2.73-6.08)**	**3.70 (2.20-6.25)**
**Sedentary job**		1.42 (0.96-2.08)	1.65 (0.99-2.77)
**Previous sedentary job**		1.11 (0.85-1.44)	1.01 (0.70-1.44)
**Outdoor job**		0.93 (0.65-1.31)	0.72 (0.45-1.17)
**Previous outdoor job**		**1.73 (1.33-2.26)**	**1.72 (1.20-2.47)**

Table 4S. Interaction term with main effect between study variables and ethnicity with advanced PCa in a logistic regression

**Variable (Base: #<non-black)**	**ISUP ≥ 4**	**ISUP ≥ 3**
**60-67#black**	0.87 (0.24-3.07)	0.94 (0.32-2.72)
**68-75#black**	0.71 (0.21-2.40)	1.34 (0.46-3.88)
**75+#black**	**0.21 (0.04-0.98)**	0.40 (0.09-1.68)
**PCa family history#black**	0.89 (0.28-2.86)	0.66 (0.24-1.85)
**STD#black**	2.25 (0.82-6.21)	**2.70 (1.10-6.65)**
**Red meat consumption#black**	**2.42 (1.02-5.74)**	**2.20 (1.03-4.72)**
**Aspirin#black**	1.49 (0.65-3.44)	0.93 (0.42-1.92)
**Erectile dysfunction#black**	0.58 (0.20-1.70)	0.70 (0.28-1.75)
**Gynaecomastia#black**	1.95 (0.39-9.78)	2.90 (0.71-11.77)
**Subsistence farming#black**	1.70 (0.68-4.28)	2.02 (0.90-4.52)
**Medium poverty rate#black**	4.11 (0.82-20.63)	1.45 (0.50-4.21)
**High poverty rate#black**	1.27 (0.47-3.48)	2.15 (0.82-5.59)
**Frontal balding pattern#black**	3.47 (0.77-15.71)	2.68 (0.74-9.64)
**Vertex balding pattern#black**	0.18 (0.03-1.13)	0.30 (0.05-1.82)
**Complete balding pattern#black**	0.80 (0.23-2.79)	1.17 (0.38-3.60)
**Hairy chest#black**	1.56 (0.42-5.79)	1.17 (0.36-3.78)
**Diabetes#black**	0.45 (0.09-2.15)	1.14 (0.29-4.50)
**Traditional Healers#black**	0.85 (0.25-2.95)	1.13 (0.37-3.39)
**Sedentary job#black**	1.66 (0.53-5.17)	**3.54 (1.23-10.14)**
**Previous sedentary job#black**	1.27 (0.54-2.95)	1.44 (0.68-3.04)
**Outdoor job#black**	0.84 (0.28-2.46)	0.41 (0.16-1.09)
**Previous outdoor job#black**	0.68 (0.29-1.61)	0.89 (0.42-1.88)
